# Effect of breastfeeding on dietary patterns in early childhood: the CoAlHaS study

**DOI:** 10.1007/s00394-026-03985-x

**Published:** 2026-05-26

**Authors:** S. Ferro-Larroy, I. Gomez-Acebo, J. Alonso-Molero, S. Llorente-Pelayo, S. Valero-Dominguez, C. Lechosa-Muñiz, M. Paz-Zulueta, M. Cabero-Perez, Trinidad Dierssen-Sotos

**Affiliations:** 1 Preventive Medicine Group, University of Cantabria, Santander, Spain; 2 IDIVAL-Valdecilla Health Research Institute, Santander, Spain; 3 CIBER Epidemiology and Public Health (CIBERESP), Madrid, Spain; 4 Department of Pediatrics, Marqués de Valdecilla University Hospital, Santander, Spain; 5 Department of Nursing, University of Cantabria, Santander, Spain; 6 RICCORS Node – Carlos III Health Institute, Madrid, Spain

**Keywords:** Breastfeeding, Child nutrition, Diet quality, NOVA classification, Ultra-processed foods

## Abstract

**Purpose:**

This study assessed the association between breastfeeding practices during the first year of life and later diet quality at ages 4 and 6 years, focusing on unprocessed/minimally processed foods (MPFs) and ultra-processed foods (UPFs).

**Methods:**

We analyzed data from 574 children participating in the CoAlHaS study, a prospective birth cohort conducted in Santander, Spain. Breastfeeding status was recorded at 6 months (exclusive, mixed or infant formula), and at 12 months (continued breastfeeding or infant formula). Dietary intake at ages 4 and 6 years was assessed using the Food Propensity Questionnaire from the ENALIA study, developed for the Spanish population of children and adolescents. Foods were classified according to the NOVA system. Logistic regression estimated odds ratios (OR) for high MPFs and UPFs intake (highest vs. lower tertiles), adjusting for sociodemographic, anthropometric, lifestyle variables, and total energy intake.

**Results:**

Exclusive breastfeeding at 6 months was associated with higher odds of high MPFs intake at age 4 (OR 2.2; 95% CI 1.14–4.34), but this was not significant at age 6 after adjustment. Conversely, exclusive breastfeeding consistently protected against high UPFs intake, remaining significant at age 6 (OR 0.46; 95% CI 0.23–0.91) in fully adjusted models. Continued breastfeeding at 12 months showed a slightly weaker protective association at age 6 (OR 0.55; 95% CI 0.31–0.97).

**Conclusion:**

Exclusive breastfeeding for 6 months may contribute to healthier dietary patterns in early childhood, particularly by reducing ultra-processed food consumption, with protective effects persisting until school age.

**Supplementary Information:**

The online version contains supplementary material available at 10.1007/s00394-026-03985-x.

## Introduction

The early childhood period represents a critical window for establishing dietary patterns that can influence health outcomes throughout life. During these formative years, children’s food preferences and eating behaviors are shaped by a complex interplay of biological, familial, and environmental factors, with potential long-term implications for nutritional status and chronic disease risk. While genetic predispositions contribute to appetite traits, early-life experiences with various tastes and flavors are crucial in promoting healthy eating habits [1, 2].

Importantly, the development of flavor perception begins in utero and continues through infancy, characterized by innate preferences for sweet and umami tastes and natural aversions to bitter and sour flavors [1]. Breastfeeding plays a fundamental role in shaping children’s dietary preferences, as maternal diet influences the flavors present in amniotic fluid and breast milk, exposing infants to a range of tastes [3, 4]. These early exposures have been linked to increased acceptance of similarly flavored foods in later childhood, contributing to healthier dietary patterns [3]. For example, infants exposed to carrot flavor prenatally or through breast milk show fewer negative responses to carrot-flavored cereal compared with those without prior exposure [4]. Such findings highlight breastfeeding’s influence beyond basic nutrition, helping explain cultural variations in food preferences and reinforcing its importance in shaping long-term dietary behaviors [4].

Beyond early flavor exposure, parental food habits and feeding strategies significantly shape children’s dietary preferences. While restrictive feeding and excessive control often have counterproductive effects, fostering unhealthy relationships with food [5], repeated positive exposure to novel foods enhances acceptance [1]. As children grow, additional external factors such as peer dynamics and food availability further influence their eating behaviors [6].

In recent years, attention has focused on the role of food processing in diet quality. The NOVA food classification system categorizes foods based on their degree of processing into four groups: unprocessed or minimally processed foods (MPFs), processed culinary ingredients, processed foods, and ultra-processed foods (UPFs) [7]. UPFs are industrial formulations containing substances not commonly used in traditional culinary preparations, such as preservatives, emulsifiers, and artificial flavors. Despite some debate about the NOVA system’s application and concerns regarding nutrient profiles and food safety [8, 9], it has become a widely used framework in nutritional epidemiology to assess the impact of food processing on health.

Studies across diverse pediatric populations consistently show dietary patterns dominated by high UPFs consumption alongside low fruit and vegetable intake [10–15]. This pattern is linked to adverse health outcomes, underscoring the need to promote healthier eating habits in early life.

The health implications of high UPFs intake during childhood are well documented. Increased UPFs consumption correlates with higher intake of energy, lipids, and sodium, while reducing protein, fiber, and micronutrient consumption [16]. Longitudinal studies further indicate that frequent UPFs intake adversely affects nutritional status and body composition over time [17] with associations to unfavorable growth trajectories and increased cardiometabolic risk factors extending into young adulthood [18, 19].

Conversely, MPFs contribute essential nutrients such as dietary fiber, vitamin D, calcium, and potassium, while generally providing lower energy and added sugars [20]. Higher MPFs intake is associated with better diet quality, reflected in reduced energy, lipid, and sodium consumption [16, 21]. Additionally, MPFs-rich diets support gut microbiota balance and anti-inflammatory responses, fostering metabolic and immune health in childhood [13].

Despite growing evidence about early dietary influences, longitudinal data assessing the evolution of food consumption from birth through early childhood remain scarce. To fill this gap, the present study examines the association between breastfeeding during the first year of life and dietary patterns at ages 4 and 6, focusing on consumption of MPFs (NOVA Group 1) and UPFs (NOVA Group 4) over a two-year period.

## Methods

### Design and setting

This work is part of the CoAlHas study (*Covid*,* Alimentacion y Hábitos Saludables*) a prospective cohort investigation conducted at the Marqués de Valdecilla University Hospital (HUMV), a tertiary-level public hospital in Santander, northern Spain. HUMV is a participant in the Baby-Friendly Hospital Initiative and manages approximately 2500 deliveries annually.

A total of 2012 children were recruited at birth across two distinct periods. The pre-pandemic cohort included 967 newborns recruited between January and August 2018, whereas the pandemic cohort comprised 1045 newborns recruited between March and December 2020. For the present study, only participants from the initial pre-pandemic cohort who completed the Food Frequency Questionnaire (*n* = 574) were included.

### Follow-up and data collection

All participants were systematically followed over their first six years of life, during which comprehensive data were collected.

#### Data collected during the first year of life

Data were obtained at three time points: birth, 6 months, and 12 months of age. At delivery, maternal age, obstetric variables (including delivery mode and Apgar scores), neonatal anthropometric measurements (weight, length, head circumference), initial feeding practices, and family socioeconomic characteristics were recorded. Follow-up visits at 6 and 12 months included reassessment of feeding practices (breastfeeding status, introduction of complementary foods) and anthropometric parameters.

##### Type of feeding: data management

The type of feeding was recorded through interviews with mothers at the time of hospital discharge and subsequently at 6 and 12 months of the child’s life.

Feeding practices were classified into three categories: breastfeeding, mixed feeding, and infant formula. At 12 months, feeding was grouped into two categories-continued breastfeeding versus infant formula only-since no infants were exclusively breastfed at this age and all breastfed children were receiving complementary foods. To allow comparison of feeding effects at 6 and 12 months, feeding at 6 months was recoded using the same dichotomous definition applied at 12 months (any breastfeeding versus formula feeding), and all analyses presented in Table 2 are based on this classification.

Breastfeeding was defined according to the World Health Organization’s (WHO) criteria for “exclusive breastfeeding”, whereby the infant receives only breast milk from birth (including expressed breast milk or milk from a wet nurse) and may also receive oral rehydration solutions, drops, or syrups (including vitamins, minerals, and medicines), but no other food or drink, including water. Mixed feeding referred to infants who combined breastfeeding with infant formula, and it referred strictly to this combination; it did not include the provision of water or any complementary foods, whereas infant formula feeding was defined as the exclusive consumption of formula milk.

Parents of these children were contacted via telephone and invited to participate in a follow-up phase of the project. This continuation involved a series of structured telephone interviews and in-person visits, targeting two key time points: when the children reached four and six years of age.

#### Follow-up data at 4 and 6 years of age

##### Dietary patterns: data management

Dietary intake data were assessed using a Food Propensity Questionnaire (FPQ) from the *ENALIA* study (*Encuesta Nacional de Alimentación en la Población Infantil y Adolescente*), a Spanish national survey designed for children and adolescents aged 6 months to 17 years [22]. The FPQ included 44 food items and 13 additional items on dietary supplements, allowing for a comprehensive dietary assessment. The Food Propensity Questionnaire (FPQ) was completed by one of the child’s parents, most often the mother, during structured telephone interviews conducted by the research team.

Subsequently, a trained team of dietitians estimated nutrient and energy intakes using updated Spanish Food Composition Tables [23, 24]. For each food item, intake was estimated by multiplying its reported consumption frequency by a standard portion size defined for the age group and the nutrient composition per 100 g of the edible part. Standard portion sizes were derived from national dietary guidelines to ensure consistency across participants. Total energy intake (TEI) was determined by summing the energy contributions of all individual food items.

Using the NOVA Food Classification System, we categorized FPQ items into two groups representing the extremes of food processing: unprocessed or minimally processed foods (NOVA 1) and ultra-processed foods (NOVA 4), following the definitions established by Monteiro et al. [7]. The specific items classified into each group are detailed in Supplementary Table 1. For each participant, we calculated the daily energy intake (kcal/day) from foods in NOVA groups 1 and 4 and determined their percentage contribution to TEI. These analyses were conducted at two time points—ages four and six years—to assess longitudinal dietary trends.

In addition to the primary exposure variable **(**type of feeding**)** and the primary outcome (dietary patterns), which are discussed in detail, the following data were gathered:

##### Sociodemographic data

###### Maternal and paternal educational level

Educational level of both parents was collected through self‑reported data and categorised into three groups: basic education, secondary education, and university education.

###### Household income indicators

Monthly income information was collected separately for both parents, using ordinal categorical income brackets ranging from less than €570 to more than €6,000 per month (including a “do not know/no answer” option). These categories were converted into approximate continuous values using category midpoints, and parental incomes were combined to derive total household income. Income per consumption unit was then calculated using the OECD‑modified equivalence scale to account for household size and composition.

##### Anthropometric data

Parental Body Mass Index (BMI) was calculated from self-reported height and weight provided at the beginning of the follow-up period (at the child’s age of 4 years). Once recorded as a continuous variable, BMI was classified into three categories for more practical stratification in subsequent analyses: Normal weight (BMI = 18.5–24.9), Overweight (BMI = 25–29.9), Obesity (BMI ≥ 30).

##### Physical activity and sedentary behaviors

Were assessed using the Camargo questionnaire [25] a validated tool designed to evaluate these behaviors in children under 10 years of age based on a seven-day recall. Weekly metabolic equivalents (METs-h/wk) were estimated for each child using the information gathered from the questionnaire. For this purpose, the metabolic equivalents for youth (METy) provided in the Youth Compendium of Physical Activities [26] were applied in combination with the reported duration of each activity. To prevent overestimation of activity time, a proportional correction was implemented whenever the total daily duration of reported physical activities exceeded five hours. This adjustment was performed using an individual correction factor, proportionally applied to each recorded activity. This method preserved the relative distribution of time among activities while ensuring the total remained within the established maximum limit. Calculations were conducted at two time points: first at 4 years of age (visit 1), and later at 6 years of age (visit 2).

### Statistical analysis

Descriptive results are presented as frequencies (percentages) or as means ± standard deviations. To compare means of total energy intake and energy from MPFs and UPFs (in kcal/day and as a percentage of total energy) between children who received any breastfeeding and those who were formula-fed at 6 and 12 months, Student’s *t*-test was used.

The association between feeding practices and dietary patterns was assessed using logistic regression. Energy intake from MPFs and UPFs (as a percentage of total energy) was categorized into tertiles. High consumption was defined as intake in the highest tertile and compared with the lower two tertiles.

Two different types of adjustment were made; Model 1 included child sex and age (in months), maternal age at delivery, maternal education and paternal education, Income per consumption unit, and total energy intake at the corresponding follow-up (4 or 6 years). Model 2 included all variables from Model 1 and was further adjusted for child birth anthropometrics, parental anthropometric data, and weekly physical activity (METs). The results are reported as crude and adjusted odds ratios with 95% confidence intervals (95% CI). A 5% significance level was used for these analyses and all reported p values are two-tailed. Two-tailed p values for linear trend tests across feeding type were calculated. All statistical analyses were conducted using Stata 18/SE software (StataCorp, College Station, TX, USA).

## Results

### Study population characteristics

Of the 967 mother–infant pairs enrolled in 2018, 62 (6.4%) could not be contacted at the 4-year follow-up in 2022, and 325 (33.6%) declined to participate. Among the remaining 580 participants (59.9%), 574 (98.9%) completed the dietary questionnaire and were included in the analysis.

The participants had a balanced sex distribution, with male infants representing 50.87% (*n* = 292). At the 4-year follow-up, children had a mean age of 54.9 months (SD 2.8), with no significant differences between infant feeding groups at 6 months (*p* = 0.102). Child BMI (mean 16.21, SD 1.61; *p* = 0.848), weekly physical activity (mean 68.14 MET-hours, SD 20.20; *p* = 0.5605), and daily screen time (mean 68.60 min, SD 46.33; *p* = 0.122) were also similar across groups. Eating location showed a comparable distribution, with most children eating at their parents’ home (50.70%) or at school (33.80%), and no significant differences between feeding patterns (*p* = 0.092).

Parental BMI did not differ by feeding group (mothers: mean 25.00, SD 5.05; *p* = 0.1486; fathers: mean 26.60, SD 4.00; *p* = 0.1186). In contrast, household socioeconomic indicators varied significantly. Families who practiced exclusive breastfeeding reported higher mean monthly income (3085.94 €) compared with those using formula (2797.28 €) (*p* = 0.0381). Economic level distribution also differed (*p* = 0.001), with low-income households more frequent in the mixed feeding (38.17%) and formula groups (38.18%), while high-income households were more common among exclusively breastfeeding families (35.98%). These patterns are summarized in Table 1.

**Table 1:** Sociodemographic and anthropometric characteristics of the study population according to infant feeding patterns at 6 months

	All participants	Exclusive breastfeeding	Mixed breastfeeding	Formula	
	Mean (SD)	Mean (SD)	Mean (SD)	Mean (SD)	*p*
*Child characteristic*					
Child´s age (months)	54.86 (2.78)	55,09 (2.66)	54.41 (2.53)	54.89 (2.94)	0.1024
Child´s BMI	16.21 (1.61)	16,23 (1.64)	16.13 (1.66)	16.24 (1.58)	0.8481
Physical activity (METs-h/wk)	68.14 (20.20)	68.00 (21.01)	69.79 (20.01)	67.48 (19.96)	0.5605
Screen time (min/day)	68.60 (46.33)	62,20 (38.17)	70.03 (50.12)	71.64 (48.78)	0.1218
Main meal location^a^	n (%)	n (%)	n (%)	n (%)	
Parents´ home	291 (50.70)	98 (59.76)	58 (44.27)	133 (48.36)	0.0916
School	194 (33.80)	45 (27.44)	47 (35.88)	100 (36.36)	
Grandparents´ home	59 (10.28)	12 (7.32)	18 (13.74)	29 (10.55)	
Others	21 (3.66)	5 (3.05)	7 (5.34)	9 (3.27)	
*Parental BMI*					
Mother´s BMI	25.00 (5.05)	24,39 (4.29)	24,93 (4.62)	25.47 (5.66)	0.1486
Father´s BMI	26.60 (4.00)	26,22 (3.61)	27,28 (4.46)	26.49 (3.99)	0.1186
*Household income* ^b^					
Total household income (€/month)	2947.17 (1278.30)	3085.94 (1176.08)	3058.86 (1500.45)	2797.28 (1213.16)	0.0381
Household income tertiles	n (%)	n (%)	n (%)	n (%)	
Low economic level ($\:\le\:$2350€/month)	193 (33.62)	38 (23.17)	50 (38.17)	105 (38.18)	0.0011
Middle economic level (> 2350€ to < 3200€/month)	193 (33.62)	63 (38.41)	33 (25.19)	97 (35.27)	
High economic level ($\:\ge\:$3200€/months)	175 (30.49)	59 (35.98)	46 (35.11)	66 (24.00)	

^a^Meal location refers to the primary setting where the child’s meals are consumed. ^b^Household income tertiles were calculated based on the distribution of the total monthly income within the current study population

Compared with those lost to follow-up during the first year, participants who completed the 4-year assessment had longer breastfeeding durations and more favourable socioeconomic indicators, including higher maternal employment rates and more advantageous pharmaceutical contribution categories. No significant differences were found in demographic characteristics, mode of delivery, or neonatal anthropometric measures. Further details are provided in Supplementary Table 2.

### Total and NOVA-categorized energy intake from ages 4 to 6 years, and associations with infant feeding type

Table 2 shows the changes in TEI and its distribution for the two NOVA food categories evaluated in this study between ages 4 and 6, as well as differences in energy intake by type of infant feeding at 6 and 12 months of age.

**Table 2:** Daily energy intake by food processing level and type of feeding at 4 and 6 years of age

Variable	Age: statistic	All participants	*N*	Continued breastfeeding at 6 months	*N*	Formula at 6 months	*N*	*p*	Continued breastfeeding at 12 months	*N*	Formula at 12 months	*N*	*p*
TEI ^a^ (kcal/day)	4 y: Mean (SD)	1396.73 (316.37)	569	1379.05 (314.27)	294	1415.68 (319.98)	275	0.169	1376.49 (310.35)	174	1405.68 (320.43)	395	0.314
	6 y: Mean (SD)	1482.34 (298.39)	452	1437.79 (292.12)	232	1530.21 (299.98)	220	0.001	1431.66 (294.16)	138	1505.24 (299.14)	314	0.016
TEI (kcal/kg/day)	4 y: Mean (SD)	79.59 (21.50)	552	78.84 (21.91)	287	80.40 (21.07)	265	0.395	78.62 (22.03)	170	80.02 (21.28)	382	0.480
	6 y: Mean (SD)	67.38 (17.41)	297	65.45 (17.64)	152	69.42 (16.99)	145	0.050	65.09 (17.94)	88	68.35 (17.13)	209	0.140
MPFs^b^ energy (kcal/day)	4 y: Mean (SD)	468.06 (108.26)	573	474.90 (109.83)	294	461.05 (106.62)	275	0.128	475.54 (111.58)	174	464.98 (106.97)	395	0.285
	%TEI (95%CI)	34.52(33.78–35.26)	573	35.53 (34.45–36.62)	294	33.47 (32.47–34.46)	275	0.006	35.48 (34.15–36.80)	174	34.12 (33.22–35.01)	395	0.097
	6 y: Mean (SD)	520.63 (114.64)	452	522.28 (104.14)	232	519.93 (124.53)	220	0.828	521.17 (99.13)	138	521.12 (120.64)	314	0.996
	%TEI (95%CI)	35.88(35.14–36.62)	452	37.16 (36.14–38.18)	232	34.55 (33.50–35.60)	220	< 0.01	37.2 (35.98–38.42)	138	35.32 (34.40-36.23)	314	0.021
UPFs^c^ energy (kcal/day)	4 y: Mean (SD)	703.71 (281.75)	573	677.58 (281.23)	294	731.81 (281.71)	275	0.022	666.01 (271.87)	174	720.43 (285.84)	395	0.034
	%TEI (95%CI)	48.82(47.90-49.75)	573	47.51 (46.15–48.86)	294	50.22 (48.96–51.48)	275	0.004	46.95 (45.24–48.65)	174	49.64 (48.54–50.75)	395	0.009
	6 y: Mean (SD)	732.17 (259.07)	452	682.31 (252.32)	232	784.65 (258.02)	220	< 0.01	678.76 (244.69)	138	755.58 (263.30)	314	0.004
	%TEI (95%CI)	48.23 (47.25–49.21)	452	46.24 (44.86–47.62)	232	50.31 (48.96–51.66)	220	< 0.01	46.20 (44.57–47.84)	138	49.11 (47.90-50.31)	314	0.007

^a^TEI: Total energy intake. ^b^MPFs (Minimally processed foods, NOVA Group 1). ^c^UPFs (Ultra-processed foods, NOVA Group 4)

TEI increased moderately between 4 and 6 years of age. Mean values rose from 1396.73 kcal/day at 4 years to 1482.34 kcal/day at 6 years, while mean TEI per kilogram of body weight decreased from 79.59 to 67.38 kcal/kg/day. At age 6, both total TEI and TEI per kilogram were lower among children with continued breastfeeding at 6 months (including exclusive and mixed breastfeeding) compared with those who were exclusively formula-fed, with significant differences observed for both metrics. Although total TEI at 6 years also differed significantly according to feeding type at 12 months, this association was not observed when energy intake was expressed per kilogram of body weight.

TEI from MPFs represented approximately one-third of total intake at both time points. A slight increase was observed between 4 and 6 years, from 468.06 kcal/day (34.52% of TEI) to 520.63 kcal/day (35.88% of TEI). In contrast, UPFs contributed nearly half of TEI, with values of 703.71 kcal/day (48.82%) at 4 years and 732.17 kcal/day (48.23%) at 6 years. Despite a modest increase in absolute intake, the relative contribution of UPFs remained largely unchanged over time.

Daily energy intake was higher among children who were formula-fed at 6 and 12 months of age, with significant differences observed at the 6-year assessment. At age 6, children formula-fed at 6 months consumed a mean of 1530.21 kcal/day (SD 299.98), compared with 1437.79 kcal/day (SD 292.12) in the continued breastfeeding group (*p* = 0.01). Similarly, those formula-fed at 12 months consumed 1505.24 kcal/day (SD 299.14), versus 1431.66 kcal/day (SD 294.16) in the continued breastfeeding group (*p* = 0.016). The proportion of total energy intake from MPFs was higher in the continued breastfeeding group, with differences that were statistically significant at both 4 or 6 years of age, regardless of whether feeding type was assessed at 6 or 12 months.

In contrast, both absolute and relative energy intake from UPFs were consistently higher among formula-fed children. At age 4, children formula-fed at 6 months consumed more energy from UPFs than children with continued breastfeeding (731.81 vs. 677.58 kcal/day; *p* = 0.022), corresponding to a significantly higher proportion of total energy intake (50.22% vs. 47.51% of TEI; *p* = 0.004). Similar differences were observed when infant feeding was assessed at 12 months (720.43 vs. 666.01 kcal/day; *p* = 0.034; 49.64% vs. 46.95% of TEI; *p* = 0.009). These differences persisted at age 6: children formula-fed at 6 months consumed 784.65 kcal/day (SD 258.02) from UPFs compared with 682.31 kcal/day (SD 252.32) in the continued breastfeeding group (*p* < 0.01), representing 50.31% versus 46.24% of TEI (*p* < 0.01). Comparable results were found for formula feeding at 12 months (755.58 vs. 678.76 kcal/day; *p* = 0.004; 49.11% vs. 46.20% of TEI; *p* = 0.007).

### Association between infant feeding practices and intake of unprocessed or minimally processed foods

The longitudinal analysis of feeding practices and their association with consumption of MPFs is presented in Table 3.

**Table 3:** Association between feeding practices and high consumption of minimally processed foods (NOVA 1) at ages 4 and 6 years

Exposure	Category	Cases/N	Visit 1 (age 4 years)						Visit 2 (age 6 years)					
			Crude model		Model 1		Model 2		Crude model		Model 1		Model 2	
			OR (95% CI)	*p*	OR (95% CI)	*p*	OR (95% CI)	*p*	OR (95% CI)	*p*	OR (95% CI)	*p*	OR (95% CI)	*p*
Type of feeding at 6 months	Infant formula	80/275	Reference		Reference		Reference		Reference		Reference		Reference	
	Exclusive breastfeeding	66/163	1.66(1.04–2.49)	0.015	1.74 (1.00-3.02)	0.049	2.22 (1.14–4.34)	0.019	1.54 (0.97–2.43)	0.066	1.21 (0.69–2.11)	0.503	1.21 (0.65–2.28)	0.694
	Mixed feeding	44/131	1.23(0.79–1.93)	0.358	1.01(0.57–1.80)	0.959	1.16(0.57–2.33)	0.684	1.59 (0.96–2.62)	0.070	0.90 (0.49–1.67)	0.742	0.89 (0.45–1.78)	0.744
	*p trend*			*0.015*		*0.062*		*0.022*		*0.051*		*0.527*		*0.560*
Type of feeding at 6 months	Infant formula	80/275	Reference		Reference		Reference		Reference		Reference		Reference	
	Continued breastfeeding	110/294	1.46(1.025–2.071)	0.036	1.34(0.84–2.14)	0.212	1.64(0.92–2.89)	0.503	1.56 (1.05–2.31)	0.028	1.08 (0.68–1.74)	0.757	1.02 (0.59-1,75)	0.949
Type of feeding at 12 months	Infant formula	126/395	Reference		Reference		Reference		Reference		Reference		Reference	
	Continued breastfeeding	64/174	1.24 (0.85–1.81)	0.256	1.23 (0.75–2.02)	0.414	1.56 (0.86–2.82)	0.140	1.11 (0.73–1.69)	0.633	0.88 (0.53–1.46)	0.619	0.90 (0.51–1.57)	0.701

^a^Model 1: Adjusted for child sex and age (in months), maternal age at delivery, maternal education, paternal education, Income per Consumption Unit and total energy intake at the corresponding follow-up (4 or 6 years)

^b^Model 2: Model 1 + childbirth anthropometrics, parental anthropometric data, and weekly physical activity (METs)

High consumption was defined as intake in the highest tertile compared with the lower two tertiles. OR = Odds Ratio; CI = Confidence Interval

At the 4-year follow-up, exclusive breastfeeding at 6 months was strongly associated with a higher intake of MPFs. In the fully adjusted model (Model 2), which included socioeconomic status, total energy intake, and anthropometric variables, children exclusively breastfed at 6 months were twice as likely to have a high MPFs intake compared with those formula-fed (OR 2.22; 95% CI 1.14–4.34).

In contrast, mixed breastfeeding showed a weaker and non-significant association (OR 1.16; 95% CI 0.57–2.33). A significant linear trend across breastfeeding categories was observed (p-trend = 0.022), suggesting a dose–response relationship after adjustment for covariates.

By the second follow-up, at 6 years of age, the association between exclusive breastfeeding at 6 months and higher NOVA 1 food intake was weaker than that observed at age 4. In the crude model, the association showed borderline statistical significance (OR 1.54; 95% CI 0.97–2.43), but it was attenuated and no longer significant after adjustment. Mixed breastfeeding at 6 months showed similar crude association (OR 1.59; 95% CI 0.96–2.62), which did not reach statistical significance. No association was observed between continued breastfeeding at 6 and 12 months and MPFs intake at 6 years after adjusting for sociodemographic factors.

### Association between infant feeding practices and intake of ultra-processed foods

Table 4 shows the association between early feeding practices and UPFs consumption.

**Table 4:** Association between feeding practices and high consumption of ultra-processed foods (NOVA 4) at ages 4 and 6 years

Exposure	Category	Cases/N	Visit 1 (age 4 years)						Visit 2 (age 6 years)					
			Crude model		Model 1		Model 2		Crude model		Model 1		Model 2	
			OR (95% CI)	*p*	OR (95% CI)	*p*	OR (95% CI)	*p*	OR (95% CI)	*p*	OR (95% CI)	*p*	OR (95% CI)	***p***
Type of feeding at 6 months	Infant formula	106/275	Reference		Reference		Reference		Reference		Reference		Reference	
	Exclusive breastfeeding	43/163	0.57 (0.37–0.87)	0.010	0.55 (0.31–0.98)	0.043	0.49 (0.25–0.95)	0.036	0.34 (0.21–0.56)	< 0.001	0.39 (0.21–0.72)	0.003	0.46(0.23–0.91)	0.026
	Mixed feeding	41/131	0.73 (0.47–1.13)	0.156	0.88 (0.50–1.57)	0.671	1.03 (0.52–2.01)	0.942	0.53(0.32–0.88)	0.015	0.83(0.45–1.54)	0.556	1.01(0.51–2.05)	0.957
	*p trend*			*0.080*		*0.050*		*0.048*		*< 0.001*				0.034
Type of feeding at 6 months	Infant formula	106/275	Reference		Reference		Reference		Reference		Reference		Reference	
	Continued breastfeeding	84/294	0.69 (0.44–0.91)	0.012	0.69 (0.43–1.11)	0.123	0.69 (0.40–1.20)	0.189	0.42 (0.28–0.62)	0.000	0.55 (0.34–0.90)	0.018	0.66 (0.38–1.16)	
Type of feeding at 12 months	Infant formula	148/395	Reference		Reference		Reference		Reference		Reference		Reference	
	Continued breastfeeding	42/148	0.53 (0.36–0.79)	0.002	0.51 (0.30–0.87)	0.014	0.55 (0.30–1.01)	0.056	0.46 (0.29–0.72)	0.001	0.50(0.30–0.84)	0.009	0.55 (0.31–0.97)	

^a^Model 1: Adjusted for child sex and age (in months), maternal age at delivery, maternal education, paternal education, Income per Consumption Unit and total energy intake at the corresponding follow-up (4 or 6 years)

^b^Model 2: Model 1 + childbirth anthropometrics, parental anthropometric data, and weekly physical activity (METs)

High consumption was defined as intake in the highest tertile compared with the lower two tertiles. OR = Odds Ratio; CI = Confidence Interval

At the 4-year follow-up, exclusive breastfeeding at 6 months was inversely associated with a high intake of UPFs. In the crude model, children who had been exclusively breastfed had significantly lower odds of high UPFs consumption compared with those who were formula-fed (OR 0.57; 95% CI 0.37–0.87). This inverse association was slightly stronger after full adjustment (OR 0.49; 95% CI 0.25–0.95), remaining statistically significant.

A significant linear trend was observed across feeding categories after full adjustment (p-trend = 0.048), indicating a dose–response relationship in which the likelihood of high UPFs intake decreased progressively from formula feeding to mixed and exclusive breastfeeding.

Continued breastfeeding at both 6 and 12 months was also associated with reduced odds of high UPFs intake in the crude analysis; however, these associations were no longer statistically significant after full adjustment.

By the 6-year follow-up, the protective association between exclusive breastfeeding at 6 months and UPFs consumption became more pronounced than at age 4. Significant associations were observed in both crude (OR 0.34; 95% CI 0.21–0.56) and adjusted analyses (Model 1: OR 0.39; 95% CI 0.21–0.72; Model 2: OR 0.46; 95% CI 0.23–0.91), with a significant linear trend (p-trend = 0.034).

Continued breastfeeding showed a similar pattern. At 6 months, a protective effect on UPFs consumption was observed in the first adjusted model (Model 1: OR 0.42; 95% CI 0.28–0.62), but this association was no longer statistically significant in the fully adjusted model (Model 2: OR 0.66; 95% CI 0.38–1.16). In contrast, continued breastfeeding at 12 months retained a significant protective effect even after full adjustment (Model 2: OR 0.55; 95% CI 0.31–0.97).

## Discussion

This study provides longitudinal evidence on the persistent effect of exclusive breastfeeding in shaping children’s dietary patterns. Breastfeeding, particularly exclusive breastfeeding at six months, emerged as a significant predictor of dietary patterns in later childhood. At age 4, children who had been exclusively breastfed showed a dual benefit: they had lower odds of high consumption of UPFs and higher odds of consuming MPFs. However, by age 6, the protective effect was maintained only for UPFs intake, suggesting that early feeding practices may have a more lasting influence on reducing consumption of highly processed foods than on promoting intake of minimally processed ones.

The inverse association between breastfeeding and UPFs consumption observed in our study is highly consistent with previous literature. Several studies have reported that longer breastfeeding duration is associated with lower energy intake from UPFs in children aged 4–7 years [11, 27, 28]. Similar associations have been reported for specific UPFs components, such as sugar-sweetened beverages [29, 30]. Notably, only two studies to date have specifically evaluated the relationship between exclusive breastfeeding and UPFs intake, both reporting findings consistent with ours [11, 27] .

In contrast, the positive association between exclusive breastfeeding and MPFs observed at age 4 was not evident at age 6. These findings support the broader hypothesis that the influence of breastfeeding on certain dietary behaviours may attenuate with age as other determinants, such as food availability, family preferences, peer influence, or food neophobia, become more influential [30–32]. However, other studies have reported a sustained positive association with MPFs consumption up to age 6 or beyond [27–29, 32, 33]. Differences in dietary assessment may partly account for these discrepancies, as several studies have examined specific MPFs subgroups-most often fruits and vegetables-rather than the whole category. Interestingly, some have found associations with vegetable intake but not with fruit consumption [30, 32–34], a pattern that could also help explain the weaker association observed in our study, given that vegetables often require greater sensory acceptance, while fruits are generally accepted regardless of early feeding practices [31].

Regarding overall dietary composition, we observed a slight increase in total energy intake between ages 4 and 6, accompanied by a decrease in energy intake relative to body weight (kcal/kg/day), a pattern consistent with previous findings in European children [35]. UPFs provided nearly half of daily energy, while MPFs accounted for approximately one-third.

From 4 to 6 years, the proportion of energy derived from UPFs decreased slightly (48.8 to 48.2%), while that from MPFs increased modestly (34.5 to 35.8%). At both time points, children who were formula-fed at 6 or 12 months consumed significantly more total energy and energy from UPFs compared with breastfed peers, with no significant differences observed in energy contribution from MPFs by infant feeding type.

To better interpret these findings, it is important to consider both the dietary patterns typically observed at these ages and the contexts in which food consumption occurs. Across diverse settings, children aged 4–6 years commonly exhibit dietary patterns characterized by low consumption of fruits, vegetables, legumes, and dairy products, together with high intakes of sugar-sweetened beverages, snacks, and other energy-dense, nutrient-poor foods [36, 37]. These patterns have been consistently documented across multiple regions, including Europe and Mediterranean countries, where ultra-processed foods often contribute between one third and one half of total daily energy intake [37, 38].

Eating location further shapes diet quality. In early childhood, most meals are consumed at home, with school representing the second most common eating setting [39, 40]. In our cohort, the majority of eating occasions took place in family settings, with 51.5% of meals consumed at the parents’ home and an additional 10.4% at grandparents’ homes, while 34.3% occurred at school and only 3.7% in other settings. As both home and school environments are generally associated with healthier food choices compared with leisure venues, food outlets, or eating “on the go” [39], this distribution may help to contextualize the dietary profiles observed and provide additional insight into the differences in UPFs intake observed between breastfeeding groups.

Published research reveals considerable variability in the intake of UPFs and MPFs among pediatric populations. This variability highlights the influence of geographical, socioeconomic, and methodological differences on dietary patterns. Our findings on UPFs consumption align with those reported in the European I.Family study [12], conducted across eight European countries, which identified a UPFs contribution of approximately 50% of total energy intake. Generally, UPFs account for 30% to 50% of total energy intake in children under 7 years. Lower UPFs intake estimates cluster around 30% or less [16, 21, 41], intermediate values range between 35 and 55% [13, 14, 42–44], while higher intakes (> 60%) have been reported in the United Kingdom [45]and the United States [20].

Regarding energy contribution from MPFs, the proportion in our cohort (35%) is notably higher than the 28.5% reported in the I.Family study [12]. Estimates of MPFs intake vary from approximately 25% or less [20, 21] to 30–40% [13], and clearly exceed 40% in Portugal and several Latin American countries [16, 37].

Our study has several limitations that should be acknowledged. First, the final sample lacks representativeness due to attrition during follow-up. Of the 967 mother–infant pairs originally enrolled, only 62.3% completed the 4-year follow-up and were included in the present analysis. Compared with those lost to follow-up, participants had higher breastfeeding rates and more favourable socioeconomic indicators. However, no significant differences were observed in key demographic variables, mode of delivery, or neonatal anthropometric measures. Prospective cohort studies present methodological challenges, particularly regarding attrition rates, which vary substantially depending on the length of follow-up and disproportionately affect disadvantaged populations [46, 47]. In our cohort, this selective retention of families with more favourable breastfeeding and socioeconomic profiles likely reduced variability in both exposure and key contextual factors.

Second, beyond the issue of generalizability, differential loss to follow-up may have introduced selection bias. Both socioeconomic status and breastfeeding are associated with children’s diet quality, and these same factors also predicted attrition in our cohort. As a result, restricting the analysis to participants who remained in the study may have introduced collider bias. This could have led to an underrepresentation of unhealthy dietary patterns in the lower breastfeeding group, thereby attenuating the observed associations. Consequently, the true influence of breastfeeding on promoting a healthier diet may be stronger than our findings suggest, and the observed estimates are likely biased towards the null [48]. To mitigate this potential selection bias, we adjusted all analyses for maternal education, a key predictor of both breastfeeding rates (the exposure) and children’s dietary patterns at age four (the outcomes, measured as MPFs and UPFs consumption). This analytical strategy helps block potential bias pathways arising from shared determinants of exposure and outcome that also influence follow-up participation.

Third, we did not collect information on parental diet, which could be a relevant predictor of children’s dietary patterns. In addition, we lacked data on intra-household food-related practices, such as which family member was primarily responsible for food preparation or whether children’s nutrition was explicitly discussed between parents. However, parental diet is likely correlated with other factors such as socioeconomic status and BMI, both of which were included in our analyses. Therefore, we expect minimal residual confounding from the lack of parental dietary data and related household food practices.

Despite these limitations, our study also presents notable strengths that reinforce the robustness of the findings. Its prospective birth cohort design allowed for detailed and temporally accurate data collection on infant feeding practices, minimizing recall bias and ensuring validity of exposure assessment. Furthermore, the study gathered comprehensive information on the child’s socioeconomic environment, enabling adjustment for multiple potential confounders. Another important strength is that dietary patterns were assessed at two time points (ages 4 and 6 years), allowing examination of whether the influence of breastfeeding on diet remains stable over time. This enhances both internal validity and the temporal relevance of our findings.

In conclusion, this longitudinal study provides further evidence that exclusive breastfeeding for 6 months is associated with healthier dietary patterns in early childhood and that its benefits may extend into the school-age years by reducing ultra-processed food intake, with no clear effect on MPFs. No benefits were observed for continued breastfeeding at 12 months. These findings support promoting exclusive breastfeeding during the first 6 months as part of public health strategies to improve childhood diet quality and reduce ultra-processed food consumption.

## Electronic Supplementary Material

Below is the link to the electronic supplementary material.

Supplementary Material 1

## Data Availability

The datasets generated and/or analysed during the current study are not publicly available due to data protection regulations, but are available from the corresponding author on reasonable request.
