# Supplementary material for: Effect of breastfeeding on dietary patterns in early childhood: the CoAlHaS study
Source: Eur J Nutr. 2026 May 26;65(4):137. doi: 10.1007/s00394-026-03985-x (PMC13212814; doi:10.1007/s00394-026-03985-x)
Supplement: Supplementary file 1 — Supplementary Material 1 [file 394_2026_3985_MOESM1_ESM.pdf]

Supplementary Table 1. Foods included in NOVA Groups 1 and 4, according to the NOVA classification system (adapted from Monteiro et al., 2019. FAO).

NOVA GROUP	DEFINITION	EXAMPLES
**GROUP 1 Unprocessed or minimally processed foods**	Unprocessed<br><br>Edible parts of plants (fruit, seeds, leaves, stems, roots, tubers) or of or from animals (muscle, fat, offal, eggs, milk), and also fungi, algae, all after separation from nature. Spring and tap water.<br><br>Minimally processed<br><br>Unprocessed foods altered by industrial processes such as removal of inedible or unwanted parts drying, powdering, squeezing, crushing, grinding, fractioning, steaming, poaching, boiling, roasting, and pasteurization, chilling, freezing, placing in containers, vacuum packaging, non-alcoholic fermentation, and other methods that do not add salt, sugar, oils or fats or other food substances to the original food. The main aim of these processes is to extend the life of unprocessed foods, enabling their storage for longer use, or to make them edible, and, often, to make their preparation easier or more diverse. Infrequently, minimally processed foods contain additives that prolong product duration, protect original properties or prevent proliferation of microorganisms.	Fresh, squeezed, chilled, frozen, or dried fruit and leafy and root vegetables; grains such as brown, parboiled or white rice, corn cob or kernel, wheat berry or grain; legumes such as beans, lentils, and chickpeas; starchy roots and tubers such as potatoes, sweet potatoes and cassava; fungi such as fresh or dried mushrooms; meat, poultry, fish and seafood, whole or in the form of steaks, fillets and other cuts; fresh, powdered, chilled or frozen eggs; fresh, powdered or pasteurized milk; fresh or pasteurized fruit or vegetable juices (with no added sugar, sweeteners or flavors); grits, flakes or flour made from corn, wheat, oats, or cassava; tree and ground nuts and other oily seeds (with no added salt or sugar); herbs and spices used in culinary preparations, such as thyme, oregano, mint, pepper, cloves and cinnamon, whole or powdered, fresh or dried; fresh or pasteurized plain yoghurt; tea, coffee, and drinking water.<br><br>Also includes foods made up from two or more items in this group, such as dried mixed fruits, granola made from cereals, nuts and dried fruit with no added sugar, honey or oil; pasta, couscous and polenta made with flours, flakes or grits and water; and foods with vitamins and minerals added generally to replace nutrients lost during processing, such as wheat or corn flour fortified with iron and folic acid.

Supplementary Table 1 (cont.). Foods included in NOVA Groups 1 and 4, according to the NOVA classification system (adapted from Monteiro et al., 2019. FAO).

NOVA GROUP	DEFINITION	EXAMPLES
**GROUP 4 Ultra-processed foods**	Formulations of ingredients, mostly of exclusive industrial use, made by a series of industrial processes, many requiring sophisticated equipment and technology (hence 'ultra-processed'). Processes used to make ultra-processed foods include the fractioning of whole foods into substances, chemical modifications of these substances, assembly of unmodified and modified food substances using industrial techniques such as extrusion, molding and pre-frying; use of additives at various stages of manufacture whose functions include making the final product palatable or hyper-palatable; and sophisticated packaging, usually with plastic and other synthetic materials. Ingredients include sugar, oils or fats, or salt, generally in combination, and substances that are sources of energy and nutrients that are of no or rare culinary use such as high fructose corn syrup, hydrogenated or unesterified oils, and protein isolates; classes of additives whose function is to make the final product palatable or more appealing such as flavors, flavor enhancers, colors, emulsifiers, and sweeteners, thickeners, and anti-foaming, bulking, carbonating, foaming, gelling, and glazing agents; and additives that prolong product duration, protect original properties or prevent proliferation of microorganisms. Processes and ingredients used to manufacture ultra-processed foods are designed to create highly profitable products (low-cost ingredients, emphatic branding), convenient (ready-to-consume) hyper-palatable products liable to displace freshly prepared dishes and meals made from all other NOVA food groups.	Many ready-to-consume products such as carbonated soft drinks; sweet or savory packaged snacks; chocolate, candies (confectionery); ice-cream; mass-produced packaged breads and buns; margarines and other spreads; cookies (biscuits), pastries, cakes, and cake mixes; breakfast 'cereals', 'cereal' and 'energy' bars; 'energy' drinks; milk drinks, 'fruit' yoghurts and 'fruit' drinks; 'cocoa' drinks; 'instant' sauces.<br><br>Many pre-prepared ready-to-heat products including pies and pasta and pizza dishes; poultry and fish 'nuggets' and 'sticks', sausages, burgers, hot dogs, and other reconstituted meat products; and powdered and packaged 'instant' soups, noodles and desserts.

Supplementary Table 2. Baseline characteristics of participants in the CoAlHas project, by follow-up status (retained vs lost to follow-up)

	Participants enrolled in 2018	Attrition group	Acceptance group	
**N (%)**	967 (100.00)	393 (40.64)	574 (59.36)	
**Children's characteristic**				
	n (%)			p
**Male Sex**	491 (50.78)	199 (50.64)	292 (50.87)	0.943
**Type of delivery**				
Eutocic	652 (67.43)	267 (67.93)	385 (67.07)	
Instrumental	79 (8.17)	35 (8.91)	44 (7.67)	0.663
Cesarean	235 (24.30)	91 (23.16)	144 (25.09)	
Missing Data	1 (0.10)	0 (0.00)	1 (0.17)	
**Type of breastfeeding**				
Exclusive breastfeeding at 6 months	247 (25.54)	83 (21.12)	164 (28.57)	
Mixed feeding at 6 months	193 (19.96)	62 (15,78)	131 (22.82)	0.002
Mixed feeding at 12 months	254 (26.27)	79 (20.10)	175 (30.49)	
	mean (SD)			
**Birth weight (g)**	3.245.76 (567.29)	3.214.15 (572.52)	3.267.44 (563.15)	0.152
**Gestational age (weeks)**	39.10 (1.91)	38.98 (1.91)	39.19 (1.91)	0.089
**Breastfeeding duration (months)**	5.91 (5.17)	5.08 (5.11)	6.46 (5.14)	<0.0001
**Maternal and Family characteristic**				
	mean (SD)			
**Maternal age (y.o.)**	33.63 (5.19)	32.82 (5.71)	34.19 (4.73)	<0.0001
	n (%)			
**Maternal smoking status**	122(12.62)	61 (15.52)	61 (10.63)	0.025
**Maternal work status**				
Unemployed	286 (29.70)	153 (38.93)	133 (23.33)	
Employed	667 (69.26)	237 (60.31)	430 (75.44)	<0.0001
Student	10 (1.04)	3 (0.76)	7 (1.23)	
**Family's pharmaceutical contribution**				
TSI1- TSI2: unemployed or pensioners	147 (15.20)	82 (20.87)	65 (11.32)	
TSI3: income < 18.000 euros per year	435 (44.98)	189 (48.09)	246 (42.86)	
TSI4: income 18.000 - 100.000 euros per year	334 (34.54)	101 (25.70)	233 (40.59)	
TSI5-NOFAR: income > 100.000 euros per year	14 (1.45)	7 (1.78)	7 (1.22)	<0.0001

TSI6: mutual insurance	26 (2.69)	6 (1.53)	20 (3.48)

Baseline data were collected at recruitment (birth) for children born between January and June 2018.
